# The Incidence of Marine Toxins and the Associated Seafood Poisoning Episodes in the African Countries of the Indian Ocean and the Red Sea

**DOI:** 10.3390/toxins11010058

**Published:** 2019-01-21

**Authors:** Isidro José Tamele, Marisa Silva, Vitor Vasconcelos

**Affiliations:** 1 CIIMAR/CIMAR—Interdisciplinary Center of Marine and Environmental Research, University of Porto, Terminal de Cruzeiros do Porto, Avenida General Norton de Matos, 4450-238 Matosinhos, Portugal; isitamele@gmail.com (I.J.T.); marisasilva17@gmail.com (M.S.); 2 Institute of Biomedical Science Abel Salazar, University of Porto, R. Jorge de Viterbo Ferreira 228, 4050-313 Porto, Portugal; 3 Department of Chemistry, Faculty of Sciences, Eduardo Mondlane University, Av. Julius Nyerere, n 3453, Campus Principal, Maputo 257, Mozambique; 4 Department of Biology, Faculty of Sciences, University of Porto, Rua do Campo Alegre, 4619-007 Porto, Portugal

**Keywords:** Indian Ocean, marine toxins, harmful algal bloom

## Abstract

The occurrence of Harmful Algal Blooms (HABs) and bacteria can be one of the great threats to public health due to their ability to produce marine toxins (MTs). The most reported MTs include paralytic shellfish toxins (PSTs), amnesic shellfish toxins (ASTs), diarrheic shellfish toxins (DSTs), cyclic imines (CIs), ciguatoxins (CTXs), azaspiracids (AZTs), palytoxin (PlTXs), tetrodotoxins (TTXs) and their analogs, some of them leading to fatal outcomes. MTs have been reported in several marine organisms causing human poisoning incidents since these organisms constitute the food basis of coastal human populations. In African countries of the Indian Ocean and the Red Sea, to date, only South Africa has a specific monitoring program for MTs and some other countries count only with respect to centers of seafood poisoning control. Therefore, the aim of this review is to evaluate the occurrence of MTs and associated poisoning episodes as a contribution to public health and monitoring programs as an MT risk assessment tool for this geographic region.

## 1. Introduction

The occurrence of Harmful Algal Blooms (HABs) in marine ecosystems can be one of the great threats to public health due to their capacity to produce marine toxins (MTs) as secondary metabolites [1,2,3,4,5,6,7,8,9,10,11,12,13,14]. MTs can be accumulated by distinct marine organisms such as fish, mollusks and crustaceans [15,16,17,18,19,20,21,22,23,24] which are the basic diet of coastal human populations. Suspected or confirmed episodes of human poisoning caused by MTs have been reported worldwide in the last century [20,21,25,26,27,28,29,30,31,32,33,34,35,36,37,38,39,40,41,42,43,44,45,46,47,48]. The occurrence of episodes of human poisoning occurs via ingestion of contaminated marine food due to the lack of monitoring programs in some countries or violations of national health authorities’ regulations imposing the closure of harvesting areas and seafoodcommercialization [18,20,26,35,39,45,47,49]. Despite the ideal environmental conditions for theformation of blooms in this geographical area, there are insufficient data related to their occurrence and toxin production [50]. This review analyses the occurrence of MTs and their producers along the African Indian and the Red Sea coasts (from Egypt to South Africa) and associated human poisoning episodes. The existence of monitoring programs of MTs will be also highlighted and finally, some suggestions for the control and prevention of marine toxins in this area will be presented.

## 2. Marine Toxins and Their Producers

Chemically, toxins can be grouped according to their polarity, lipophilic and hydrophilic. Concerning MT monitoring, analysis and quantification methods in seafood are described in Table 1, including bioassays, immunoassays, and analytical chemistry methods. The bioassay methods (Mouse Bioassay (MBA), Rat Bioassay (RBA)) are no longer in use due to ethical reasons according to Directive 86/609/EEC [51] and procedural variation [52] (e.g., use of different extraction solvents and consequently shortcomings). Chemical methods, mainly liquid chromatography coupled to mass spectrometry, are considered as the most promising since they are fully validated and standardized to replace bioassays in many organizations worldwide. Further information related to each toxin group such as syndromes, producers, common vectors, symptoms, detections methods in seafood, limit of detection (LOD) and quantification (LOQ) and permitted limit used in some parts of the world is also described in Table 1.

### 2.1. Lipophilic Toxins

Lipophilic toxins are lipid soluble toxins and this group comprises okadaic acid (OA), ciguatoxins (CTX), cyclic imines (CIs) [spirolides (SPXs), gymnodimines (GYMs), pinnatoxins (PnTXs) and pteriatoxins (PtTXs)], brevetoxins (PbTxs), pectenotoxins (PTXs), yessotoxins (YTXs) and azaspiracids [AZAs], Table 1.

#### 2.1.1. Okadaic Acid and Analogs

**Figure 1:**
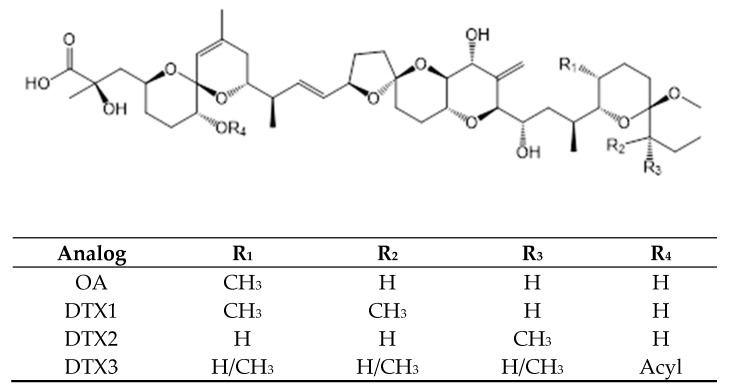
Chemical structure of OA and main derivatives [DTX1, DTX2, and DTX3].

Okadaic acid (OA)and their analogs, dinophysistoxins-1, -2 and -3 (DTXs) (Figure 1), are polyethers produced by dinoflagellates: *Prorocentrum* spp. [8], *Dinophysis* spp. [2,6,9,10,15,53,54] and *Phalacroma rotundatum* [55] (Table 1).These polyethers are frost-resistant and heat-stable and consequently, their toxicity is not affected by the cooking procedures in water (they are stable at <150 °C) [56]. The OA group is responsible for the diarrheic shellfish poisoning syndrome (DSP), with OA being the main representative of DSP toxins. Okadaic acid (OA)and its analogs act as inhibitors of the serine/threonine phosphoprotein phosphatases 1,22B,4,5 types [57,58].

#### 2.1.2. Ciguatoxins

**Figure 2:**
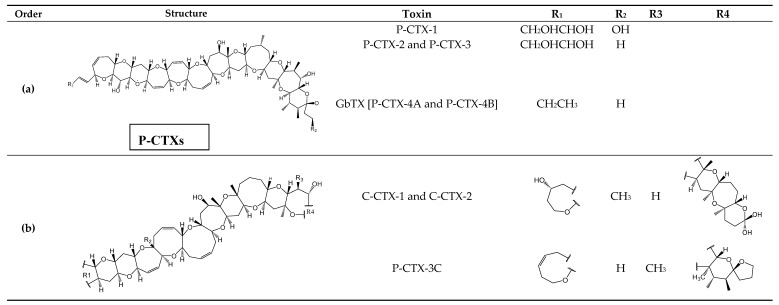

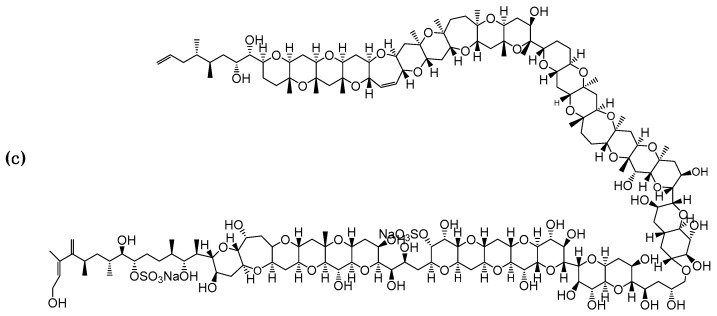
Chemical structure of major CTXs analogs from Pacific (P-CTXs) (**a**) and Caribbean (C-CTXs) (**b**) regions. The major CTXs from Indian region (I-CTXs) have a similar structure with C-CTX-1. (**c**) Chemical structure of maitotoxin (MTX).

Ciguatoxins (CTXs) (Figure 2A) are a group of toxins produced by tropical and subtropical dinoflagellates species: *Gambierdiscus toxicus* and *Fukuyoa* spp. [59,60] (Table 1) mainly found in the Pacific, Caribbean and the Indian Ocean regions [P-CTX, C-CTX and I-CTX, respectively]. CTXs are lipid-soluble polyethers with 13-14 rings fused by ether linkages into a rigid ladder-like structure [60]. To date, the structures of20 P-CTXs, 10 C-CTXSand 4 I-CTXs analogs have been fully identified and the most reported include P-CTX-1, P-CTX-2, P-CTX-3, P-CTX-3C [61,62,63,64,65,66,67], gambiertoxin [GbTXs, namely, P-CTX-4A and P-CTX-4B] [68], C-CTX-1, C-CTX-2 [67,69], I-CTX-1, I-CTX-2, I-CTX-3 and I-CTX-4 [70,71] mostly in predatory fish and gastropods [20,21,23,66,69,72,73,74]. The major analog of each group of CTXsis P-CTX-1. C-CTX-1, C-CTX-2, I-CTX1, and I-CTX-2. The chemical structure of the last two (I-CTXs) have the same molecular weight and similar structures as C-CTX-1 [62,67,70,71]. CTXs are odorless and tasteless heat-stable molecules and are not affected when subjected to water cooking, freezing and acid or basic conditions, though they suffer structural alterations by oxidation [60]. CTXs and Maitotoxin (MTX) (Figure 2B) (produced by *Gambierdiscus* spp. [68]) were the first group of toxins reported to be responsible for ciguatera shellfish poisoning (CFP) [23]. The mechanism of action of CTX and analogs is to elevate calcium ion concentration and activate non-selective cation channels in cells causing neurologic effects in humans [75].

#### 2.1.3. Cyclic Imines 

**Figure 3:**
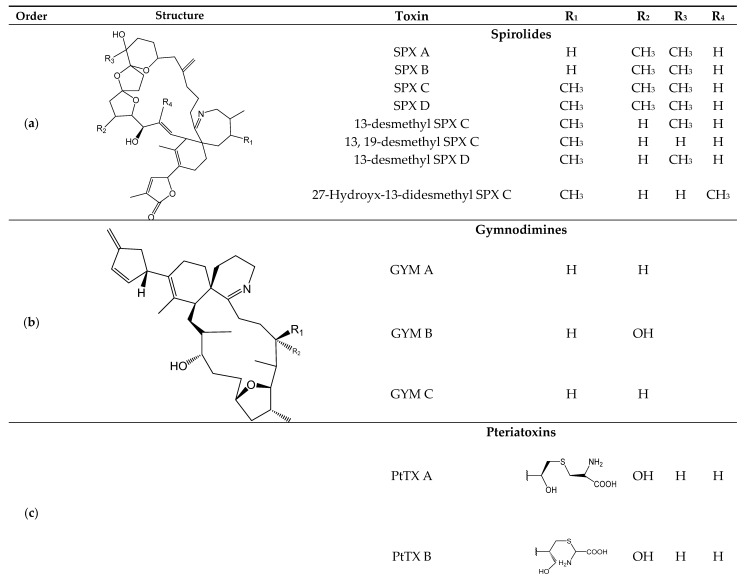

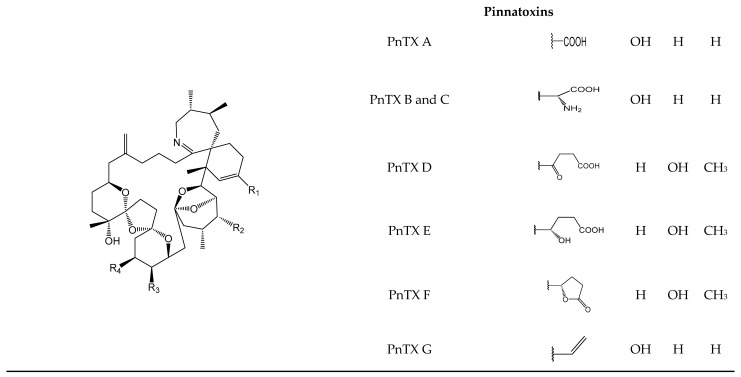
Chemical structures of CI (SPXs (**a**), GYMs (**b)**, PnTXs (**c**), and PtTXs (**c**),) and Silva et al. [79,83,84,85,86].

Cyclic imines (CI) (Figure 3) are toxins produced by dinoflagellates: SPXs: *Alexandrium* spp. [1,76], GYMs: *Gymnodium* spp. [77], PnTXs: *Vulcanodinium rugosum* [78] and PtTXs: biotransformation from PnTXs via metabolic and hydrolytic transformation in shellfish [1,5,77,78,79] (Table 1). CIs are a heterogenous group composed ofspirolides (SPXs), gymnodimines (GYMs), pinnatoxins (PnTXs) and pteriatoxins (PtTXs) and more than 24 structural analogs have been described to date [80].

Regarding chemical properties, these toxins are a group of macrocyclic compounds that have in common an imine functional group and spiro-linked ether moieties in their structure [80]. They are colorless amorphous solid macrocyclic compounds with imine and spiro-linked ether moieties [80], considerably soluble in organic solvents such as methanol, acetone, chloroform and ethyl acetate [5,80]. CIs are neurotoxins and actby inhibiting the nicotinic and muscarinic acetylcholine receptors (mAChR and nAChR, respectively) in the nervous system and at the neuromuscular junction [81]. CI bioactivity seems to depend on the imine functional group since the hydrolysis of spirolides A–D produce spirolide E and F with a keto-amine structure that is fully inactive [81]. To date, there are no regulations for CIs and no common symptoms can be recognized [82].

#### 2.1.4. Brevetoxins

**Figure 4:**
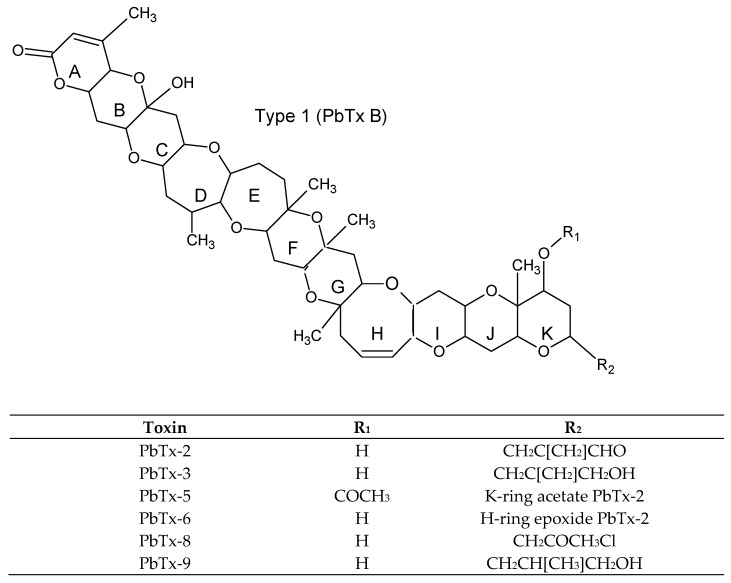

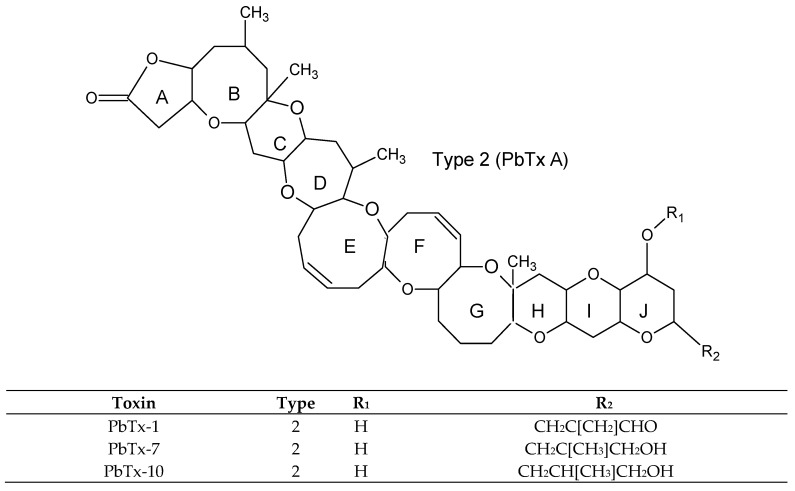
Chemical structures of the main group of PbTxs (PbTxs-A and PbTxs-B). The capital letter A in first ring indicates type A and type B (also called type 1and type 2, respectively [4]). These rings contain lactone group that is most important for the toxin activity.

Brevetoxins (PbTxs) (Figure 4) are cyclic polyethers produced by dinoflagellates: *Karenia* spp. [4,16,87] (Table 1). There are two known types of BTXs, named type A and type B (also called type 1(PbTx-1) and type 2 (PbTx-2), respectively). The difference between two types of PbTxs consists in a few transfused rings that are ten for PbTx-1 and eleven for PbTx-2. The main analogs include PbTx-3, PbTx-6, PbTx-9, PbTx-B1, PbTx-B2, S-desoxy-PbTx-B2, PbTx-B3, PbTx-B4, and PbTx-B5 [44,88,89,90,91,92,93,94]. PbTxs are lipid-soluble cyclic polyether consisting of 10 to 11 transfused rings [95], stable and resistant to heat and steam autoclaving [96]. PbTxs cause neurotoxic shellfish poisoning (NSP) and actby binding with high affinity to receptor site 5 of the voltage-gated sodium channels (Na_V_) in cell membranes, and lactone is important for the toxin activity [97]. PbTxs are regulated in USA [98], New Zealand, and Australia [99,100] (Table 1).

#### 2.1.5. Pectenotoxin Group

**Figure 5:**
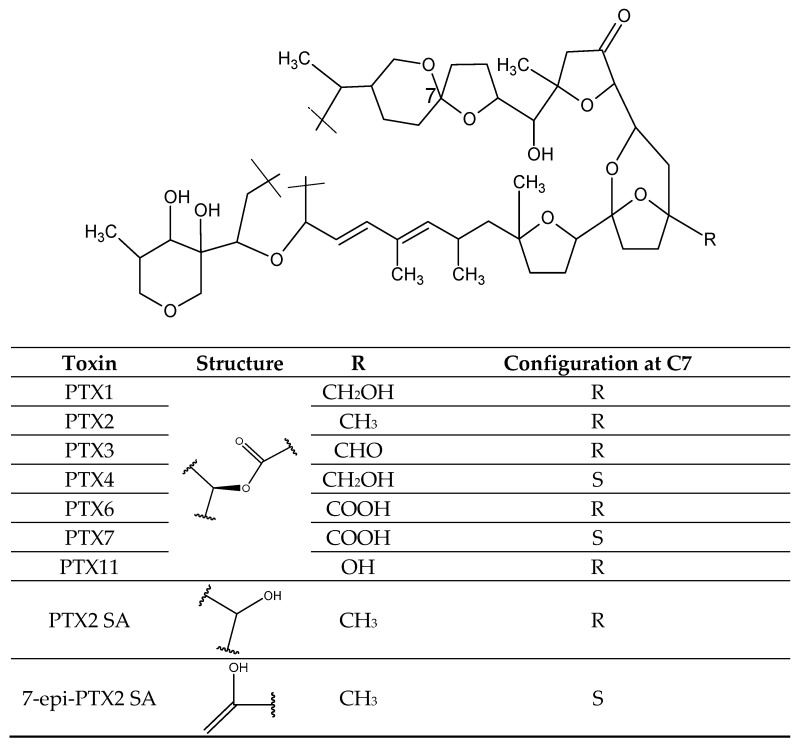
Chemical structures of main pectenotoxins.

Pectenotoxins (PTXs) (Figure 5) are lipophilic polyethers produced by several dinoflagellate species [101] (Table 1). They contain spiroketal, bicyclic ketal, cyclic hemiketals, and oxolanes in their structure. To date, more than 15 PTX analogs have been documented and many are derived through biotransformation of PTX2 in marine organism metabolism such as bivalve mollusks [102]. The most reported analogs include PTX1, *epi*-PTX1, PTX2, PTX2 *seco* acid (PTX2 SA), 7-*epi*-PTX2 *seco* acid (7-*epi*-PTX2 SA), PTX3, PTX4, PTX6, *epi*-PTX6, PTX7, PTX11 (34S-hydroxy-PTX2) [6,101,103,104,105]. PTXs are heat-stable and unstable under alkaline conditions [103]. PTX and analogs alter actin-based structures [103,106] causing cell death and apoptosis [107]. PTXs co-occur with the OA—group and contribute to DSP in humans [108].

#### 2.1.6. Yessotoxins

**Figure 6:**
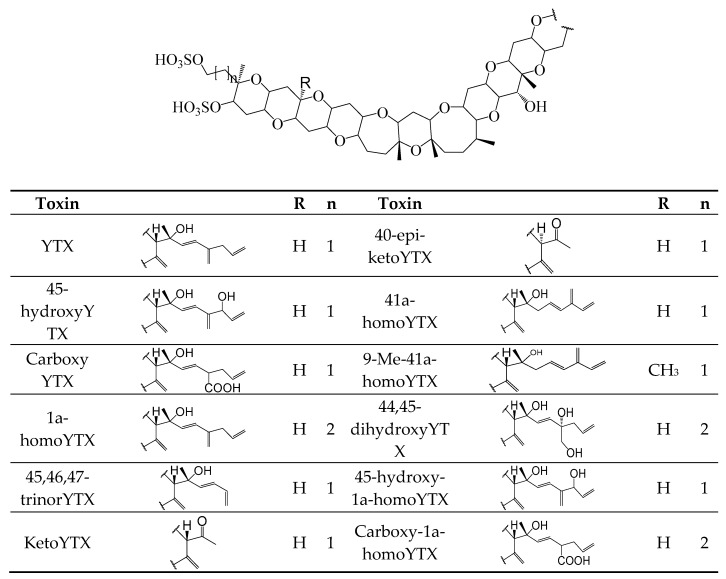
Chemical structures of YTXs n corresponds to the number of methyl groups in the molecule.

Yessotoxins (YTXs) (Figure 6) are produced by dinoflagellates species: *Protoceratium reticulatum* [4,109], *Lingulodinium polyhedral* [4] and *Gonyaulax polyhedra* [4] (Table 1). They are a heat-stable polyether, with eleven transfused ether rings, an unsaturated side chain, and two sulfate esters [110]. To date, more than 90 YTX analogues have been isolated [102] and only YTX, 45-hydroxyYTX, carboxylic, 1a-homoYTX, 45,46,47-trinorYTX, ketoYTX, 40-*epi*-ketoYTX, 41a-homoYTX, 9Me-41a-homoYTX, 44,55-dihydroxyYTX, 45-hydroxy-1a-homoYTX, carboxy-1a-homoYTX [111] have been fully identified [111]. The mechanism of action of YTX and their analogs is not fully understood; however, they are involved in phosphodiesterase activation [112] and modulation of calcium migration at several levels [113], alteration of protein disposal [114], cell change shape [115], apoptosis and cell death [116]. To date, there are no reports of human illness associated with YTXs [111].

#### 2.1.7. Azaspiracids

**Figure 7:**
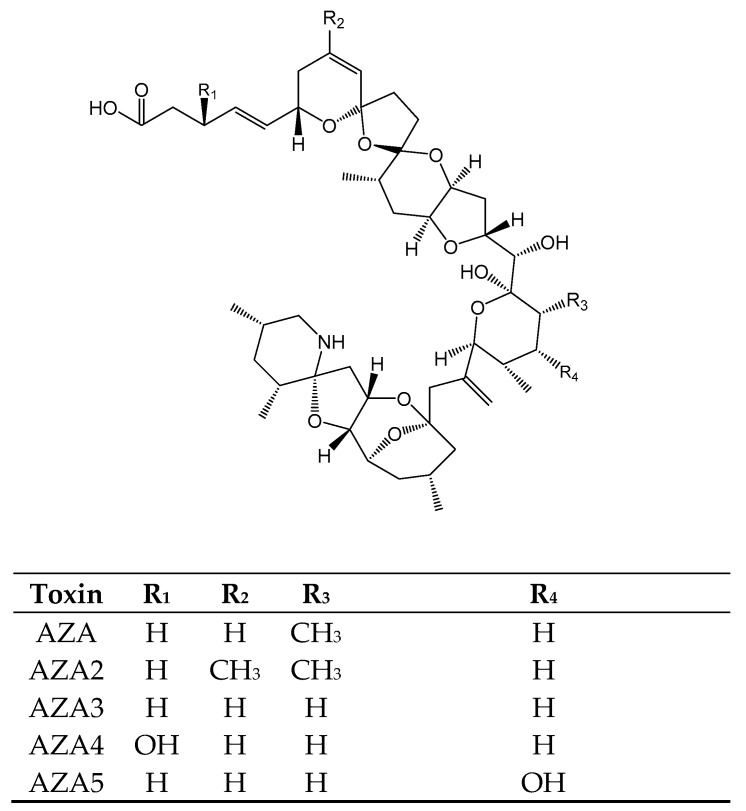
Chemical structure of AZAs.

Azaspiracids (AZAs) (Figure 7) are toxins produced by dinoflagellates: *Azadinium spinosum* [117] and *Protoperidinum crassipes* [118] (Table 1). They are colorless, odorless and amorphous solids of toxins containing a heterocyclic amine, a unique tri-spiro-assembly and an aliphatic carboxylic acid in their structures [117,119,120,121,122,123,124]. Around 21 compounds of AZAs are well known and documented [117,119,120,121,122,123,124] of which AZA, AZA2, AZA3, AZA4, and AZA5 are the most prevalent ones based on occurrence and toxicity in humans. AZAs are responsible for the AZP syndrome (Table 1) and their mechanism of action is the inhibition of hERG voltage-gated potassium channels [125].

### 2.2. Hydrophilic Toxins

Hydrophilic Toxins are polar soluble compounds and they include domoic acid (DA) and analogs, Paralytic Shellfish Toxins (PSTs), tetrodotoxins (TTXs) and palytoxins (PlTXs).

#### 2.2.1. Domoic Acid and Analogs

**Figure 8:**
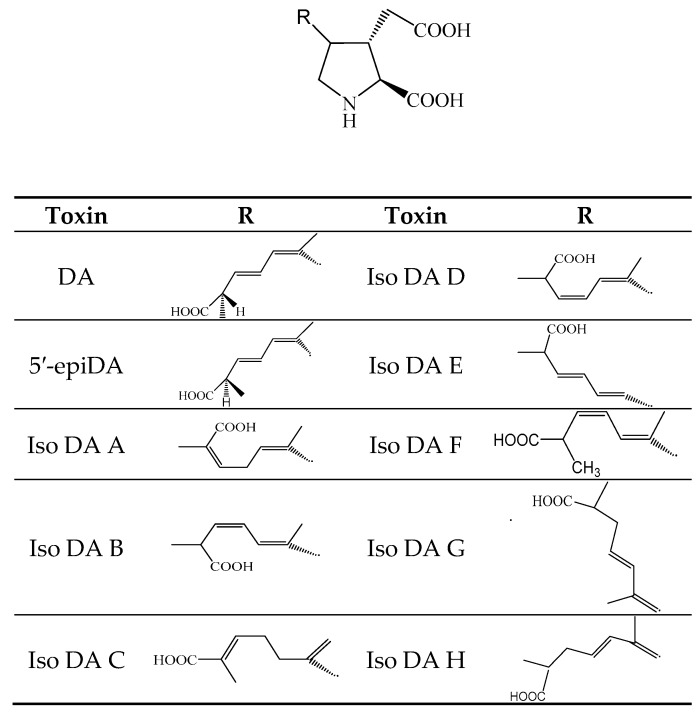
Chemical structure of DA and analogs.

Domoic acid (DA) (Figure 8) and analogs are polar cyclic amino acid toxins of diatom origin *Pseudo-nitzschia* spp. [126] and red algae: *Chondria armata* [127] (Table 1). They present three carboxylic acid groups and the most reported DA analogs include *epi*-domoic acid (*epi*-DA), domoic acid C5′-diastereomer and isodomoic acids A, B, C, D, E, F, G and H [iso-DA A-H] [128,129]. DA is the representative molecule of the DA-group that is responsible for amnesic shellfish poisoning (ASP) syndrome [130]. The characteristic symptomology of ASP is detailed in Table 1.

#### 2.2.2. Paralytic Shellfish Toxins.

**Figure 9:**
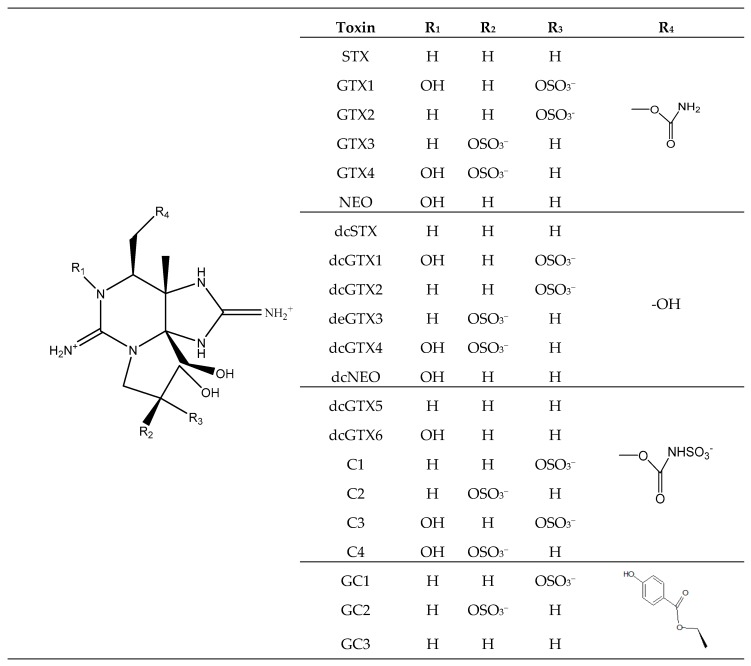
Chemical structures of STX group.

Paralytic shellfish toxins (PSTs) (Figure 9) are water-soluble tetrahydropurine toxins produced mainly by dinoflagellates *Alexandrium* spp. [2,3,7], *Gymnodinium catenatum* [3], *Pyrodinium bahamense* [3] and by cyanobacteria *Trichodesmium erythraeum* [131] except M (Figure 9) toxins that are *Mytilus* spp. metabolism products [132]. This group is composed of several analogs and they are prone to various conversions depending on pH (Figure 9), being divided into several groups: carbamoyl (saxitoxin (STX), neosaxitoxin (NeoSTX) and gonyautoxins (GTX1-4)) decarbamoyl [dc-](dcSTX, dcNeoSTX, dcGTX1-4), Nsulfo-carbamoyl [GTX5-6, C1-4], hydroxylated saxitoxins [M1-4] [133,134,135] and benzoyl toxins (GC1-3) [135]. Their heat stability is pH dependent (except for Nsulfo-carbamoyl components) [136]. STX and analogs act by binding to Nav and consequently blocking ion conductance in nerves and muscles fibers leading to paralysis [137]. Symptoms resulting from PSTs poisoning are described in Table 1.

#### 2.2.3. Tetrodotoxins

**Figure 10:**
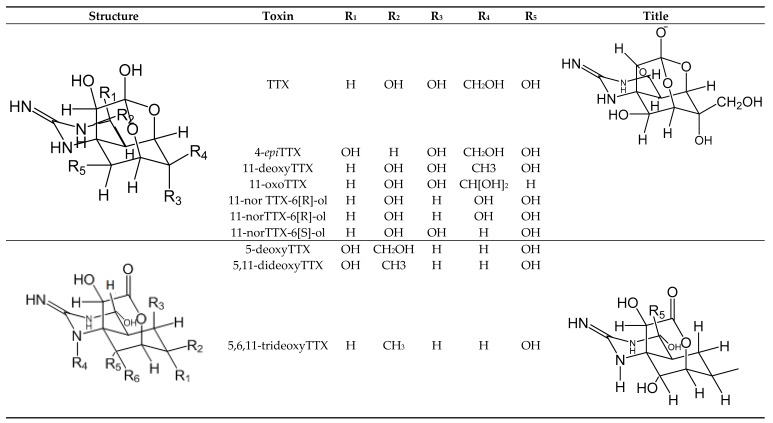
Chemical structure of TTX and their main analogues.

Tetrodotoxins (TTXs) (Figure 10) are toxins produced by bacteria in marine environments: *Serratia marcescens*, *Vibrio* spp. [83], *Aeromonas* sp. [138], *Microbacterium arabinogalactanolyticum* [139], *Pseudomonas* sp. [140], *Shewanella putrefaciens* [141], *Alteromonas* sp. [142], *Pseudoalteromonas* ssp. [143], and *Nocardiopsis dassonvillei* [144] (Table 1). They are colorless, crystalline-weak basic compounds with one positively charged guanidinium group and a pyrimidine ring [145,146]. TTXpoisoning has been recognized since ancient Egyptian times [42]. To date, TTX is considered an extremely potent emergent toxin in the Atlantic Ocean [83] and acts by binding to Nav on the surface of nerve cell membranes blocking the cellular communication and causing death by cardio-respiratory paralysis [147]. Several poisoning incidents have reported in Asia [Japan is the most affected country] [148], the Mediterranean Sea and the Indian Ocean [35]. TTX is usually concentrated in the ovaries, liver, intestines, and skin ofits principal vector [puffer fish] [42]. To date, the structures of 26 analogs of TTX have been fully elucidated but their relative toxicity and occurrence are not yet fully known [145,146] except for 12compounds, namely, TTX, 11-oxoTTX, 11-deoxyTTX, 11-norTTX-6[R]-ol, 11-norTTX-6[S]-ol, 4-epiTTX, 4,9-anhydroTTX, 5,6,11-trideoxyTTX. [131], 4-CysTTX, 5-deoxyTTX, 5,11-dideoxyTTX, and 6,11-dideoxyTTX [149,150,151,152]. 

#### 2.2.4. Palytoxin

**Figure 11:**
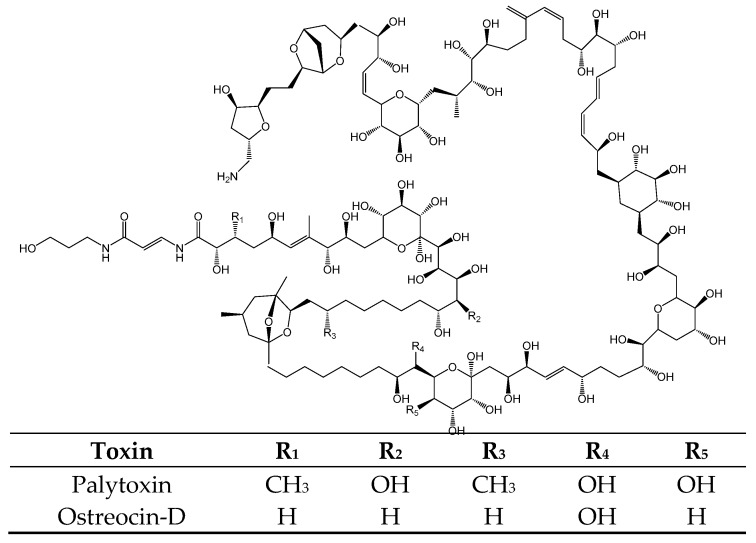
Chemical Structure of PlTXs [PTX and Ostreocin-D].

Palytoxin (PlTX) and its derivatives (Figure 11) are toxins produced by marine zoanthids *Palythoa* spp., dinoflagellates: *Ostreopsis ovata*. [153,154,155] and possibly by cyanobacteria: *Trichodesmium* sp. [156] (Table 1). These polyhydroxylated toxins have both lipophilic and hydrophilic properties [157] with a partial unsaturated aliphatic backbone containing cyclic ethers, 64 chiral centers, 40–42 hydroxyl and 2 amide groups [157]. Among PlTX analogs, known are: isobaric PlTX, ostreocin-D, ovatoxin [a to f], mascarenotoxins, ostreotoxin-1 and 2, homopalytoxin, bishomopalytoxin, neopalytoxin, deopalytoxin and 42-hydroxypalytoxin and their molecular weights range from 2659 to 2680 DA [158,159,160]. PlTX and analogs act on Na^+^, K^+^ -ATPase pumps molecules in the cell membrane [161] and the loss of intracellular contents into the blood plasma and consequent injury causing rhabdomyolysis, among other signs, are the most reported as signs of PlTX poisoning [161]. 

### 2.3. Marine Cyanotoxins

Most marine toxins reported are produced mainly by microalgae (composed basically by dinoflagellates, diatoms, and marine bacteria), while cyanobacteria are reported as toxin producers in fresh, brackish waters and terrestrial habitats. Recently, cyanotoxins typical from freshwater have been identified in the marine environment [162]. Thus, this section will be focused on the description of the most reported marine cyanotoxins involved in seafood poisoning, their producers and mode of action (Table 1).

**Figure 12:**
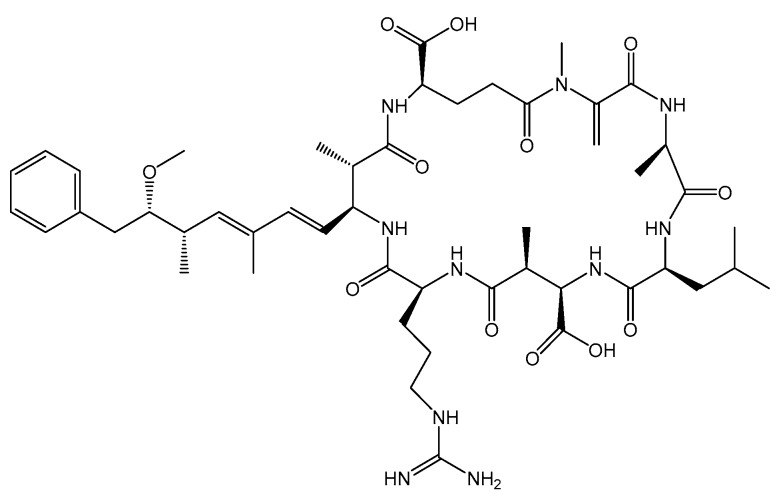
Chemical structure of MC.

One of the most relevant groups of marine cyanotoxins is themicrocystin group (MCs) [163] (Figure 12). MCs are produced by cyanobacteria of genus *Pseudoanabaena, Phormidium, Spirilia* [164], *Leptolyngbya, Oscillatoria, Geitlerinema* [165], *Trichodesmium* [166] and *Synechococcus* [167] and their occurrence have been reported in many parts of the world, namely: the central Atlantic coast of Portugal [168], Canary Islands Archipelago [166], Brazilian coast [169], Amvrakikos Gulf (Greece) [167] and Indian Ocean [170]. To date, MCs is regulated in freshwater habitats but should be extended to the marine environments since there are reports of these hepatotoxins in marine environments [162]. 

**Figure 13:**
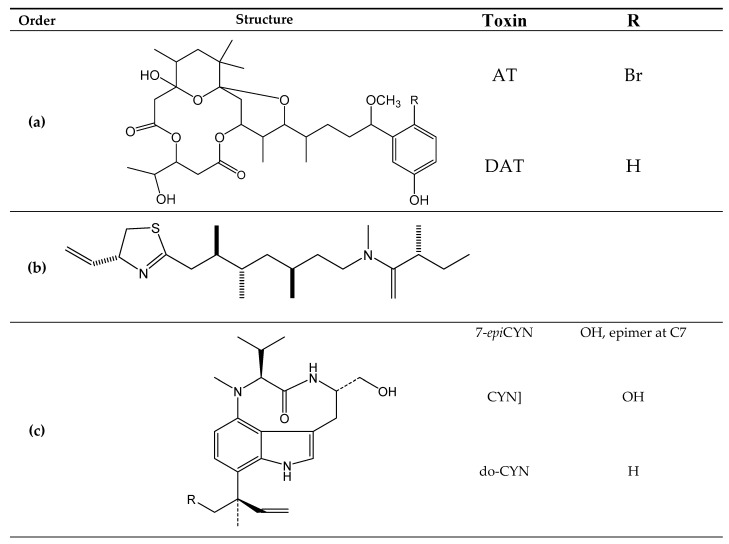

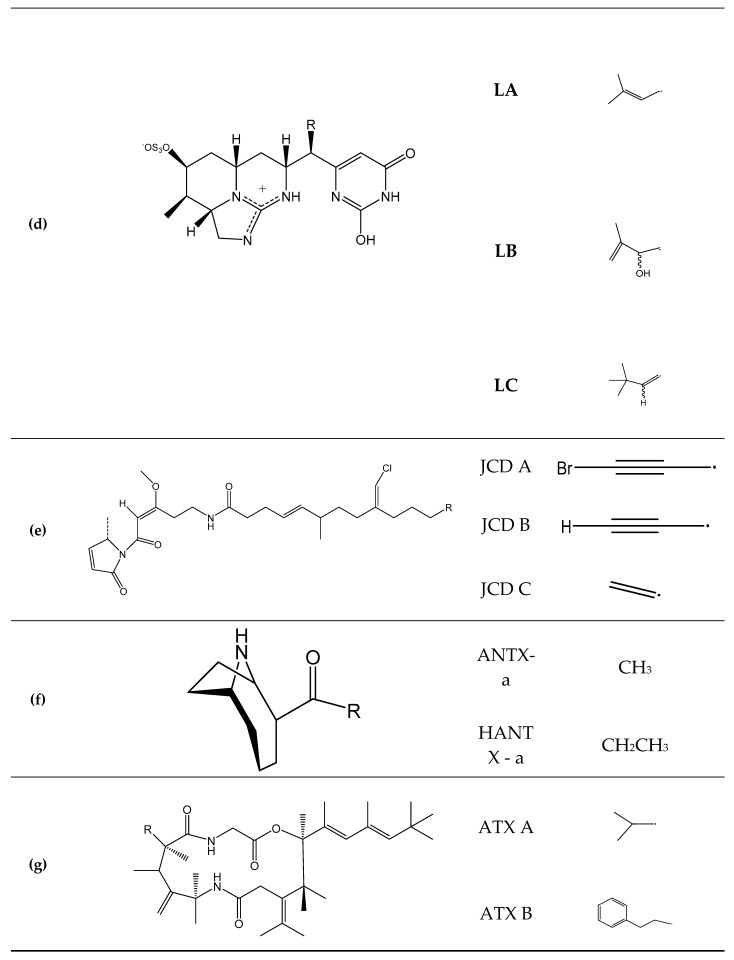
Chemical structures of Aplysiatoxin (AT) and Debromoaplysiatoxin (DAT) (**a**); kalkitoxins (KTX) (**b**); lyngbyatoxins A, B and C (LA, LB and LC) (**c**); cylindrospermopsins (CYN) (**d**); jamaicadimes (JCD) (**e**); anatoxin-a (ANTX) and homoanatoxin-a (HANTX) (**f**) and antillatoxins (ATX) (**g**).

Other reported marine cyanotoxins [in parenthesis is indicated their producers] (Figure 13) are aplysiatoxin (AT) [171] (Figure 13a), debromoaplysiatoxin (DAT) [171] (Figure 13) (algae *Gracilaria coronopifolia* [172] and cyanobacteria *Lyngbya majuscule* [171]), kalkitoxin (KTX) (cyanobacteria *Lyngbyamajuscula* [173]) (Figure 13b), lyngbyatoxins (LA, LB and LC) (cyanobacteria *Lyngbya majuscule* [174]) [Figure 13c], cylindrospermopsins (CYNs) (cyanobacteria *Cylindrospermopsis raciborskii* [175]) (Figure 13d), jamaicamides (JCDs) (Cyanobacteria *Lyngbya majuscule* [176]) (Figure 13e), anatoxins (ANTX) (cyanobacteria *Hydrocoleum lyngbyaceum* [177]) [178] (Figure 13f) andantillatoxins (ATX) (cyanobacteria *Lyngbya majuscule* [179]) (Figure 13g). The mechanism of action anddetection methods are presented in Table 1.

Recent studies indicate Homoanatoxin-a (HANTX, a derivative of anatoxin-a) produced by the cyanobacteria *Hydrocoleum* sp. and *Trichodesmium* sp. which co-occur with *G. toxicus*, may be the causative toxin of CFP [43] (rather than CTXs). This evidence suggests further studies to clarify marine cyanotoxins responsible for CFP and their mechanism of action [178]. The reports of seafood poisoning involving marine cyanotoxins are very scarce and consequently, there is no specific symptomology that can be related to marine cyanotoxin human poisoning.

## 3. Incidence of Harmful Algal Blooms MarineToxins and Consequent Poisoning Incidents along African Indian and the Red Sea Coasts 

**Figure 14:**
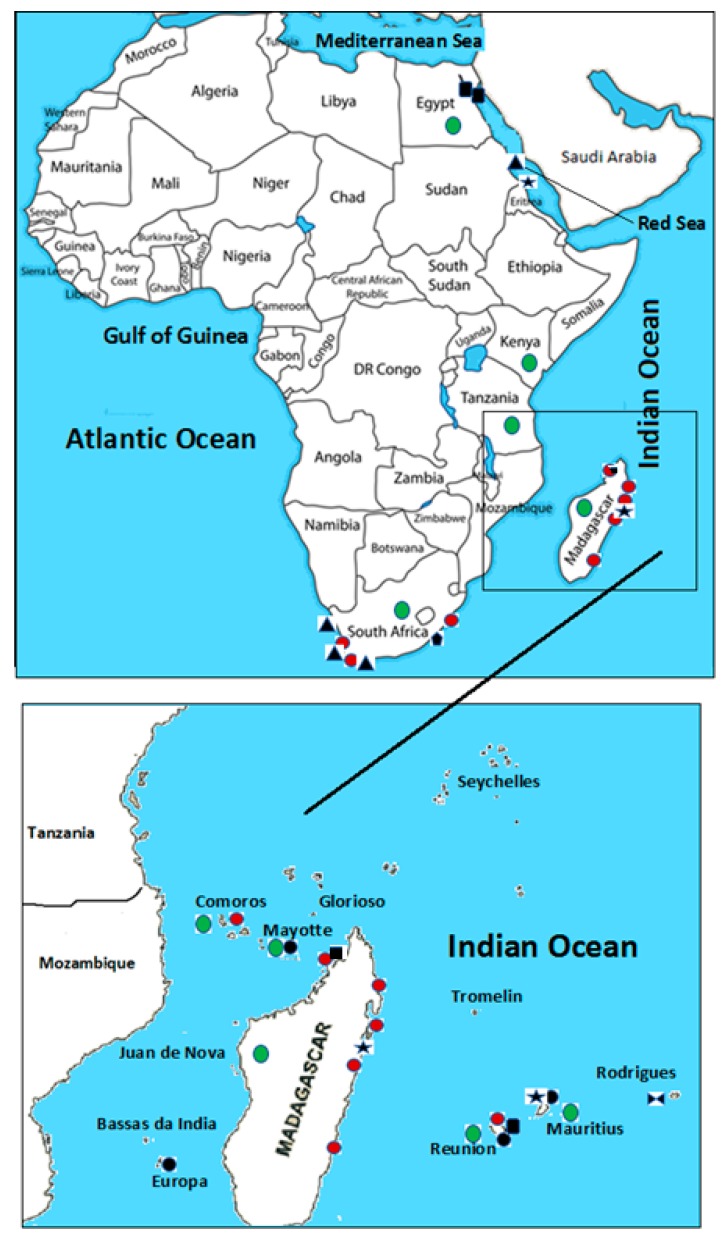
Map of the incidence of marine toxins (MT) along African countries of the Indian Ocean and the Red Sea, from EgypttoSouth Africa and nearby islands. Red circles [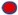]—confirmed or suspected seafood poisoning episodes caused by MT; green circles [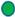]—MT or Harmful Algal Blooms monitoring programmes or Centers of seafood poisonings; 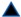
—Saxitoxins group; 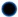
—Okadaic Acid group; 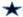
—Ciguatoxin group; 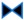
—Palytoxin group; 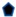
—Domoic Acid group and 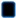
—Tetrodotoxin group.

The main geographical focus of this review is the African Indian and the Red Sea coasts, including surrounding islands (Figure 14). The marine environment of this area is understudied due to a lack of monitoring infrastructure. There is a high rate of poverty in local communities, and the local population is vulnerable to natural disasters [including HABs, tropical storms]. The exponential increase in population accompanied by industrialization and climate change contributes to eutrophication in coastal areas [295,296]. This study area is characterized as subtropical to tropical climate with a water temperature above 20 °C [297]. Eutrophication and the transportation of cysts [through maritime traffic] are considered the main factors contributing to large phytoplankton blooms, including those comprised of HAB species and/or pathogenic bacteria [295,296]. Countries with monitoring programs of marine environments related to control of seafood poisoning are listed in Table 2. A few of these programs have noted the presence of MTs (Figure 14) and HAB species [dinoflagellates, cyanobacteria, diatoms], some of which [HAB species] were detected/confirmed by microscopic techniques and some confirmed by partial 16 S rRNA genes analysis [12,13,298,299,300,301,302,303,304,305,306,307,308,309,310,311,312,313,314,315,316,317,318,319,320,321,322,323].

### 3.1. South Africa 

The occurrence of species of phytoplankton including MTs-producing HABs has been reported in coastal waters of South Africa through scientific reports and environmental monitoring programmes since 2011 [324]. Reported producer species include cyanobacteria (*Microcystisaeruginosa, Oscillatoria* sp., *Trichodesmium* sp.), dinoflagellates (*Dinophysisacuminata, D. rotundata*, *Alexandrium catenella, A. minutum*, *Gymnodinium* sp., *Prorocentrum* sp., *Gambierdiscustoxicus*, *Ostreopsis siamensis, O. ovata, P. lima, P. concavum*), diatoms (*Pseudo-nitzschia multiseries*) [19,305,309,315,331,332,333] and bacteria (*Vibrio parahaemolyticus*) [298]. Seafood poisoning cases were also reported in South Africa caused by PSTs, DSPs, PlTXs and GYM [19,216,309,334] (Table 3) after the consumption of mussels (*Donax serra*, *Perna perna* and *Chloromytilus meridionalis*) (Table 4) [37]. To minimize seafood poisoning by MTs, South Africa has implemented, through the Department of Agriculture, a program for MT monitoring in molluscan shellfish on all coasts (South African Molluscan Shellfish Monitoring and Control Programme) [324] (Table 2). This program was created based on the regulations of the European Commission (EC) Regulation, namely: Commission Regulation (EC) No 2074/2005, No 853/2004 and No 15/2011 where limit values are described for MTs and analytical techniques are advised to monitor shellfish [324]. 

Due to the absence of legislation regarding CTXs, currently, there is an absence of monitoring programs regarding this group in South Africa.Since the Indian Ocean is considered an endemic site of CTXs, this is a matter of major importance.

### 3.2. Mozambique

Studies related to HAB occurrence in Mozambique are very scarce and the few published works indicate the occurrence of dinoflagellates of the genus *Alexandrium* [313] and species of cyanobacteria (*Phormidium ambiguum, Lyngbya majuscula,* and *Lyngbya* cf. *putealis*) [307]. To date, due to the absence of a Monitoring Program and trained health staff to recognize specific symptoms of seafood poisoning in humans, there are no records of published data of MT occurrence or reports of seafood poisoning cases in this country.

### 3.3. Tanzania 

Published studies indicate the occurrence of cyanobacteria, namely: *Pseudanabaena* sp., *Spirulina labyrinthiformis*, *Spirulina* sp., *Leptolyngbya* sp., *Phormidium* sp., *Oscillatoria* sp., *Lyngbyaaestuarii, Lyngbya* sp., *Lyngbya majuscula*, *Nodularia* sp., *Synechococcus* sp., *Microcystis* sp.; Dinoflagellates: *Gambierdiscus toxicus*, *Procentrum* sp. and diatoms: *Pseudo-nitzschia* sp., *Pseudo-nitzschia pungens*, *P. seriata* and *P. cuspidate* [335,336,337,338,339,340,341]. Data related to MTs and seafood poisoning episodes are very scarce in Tanzania. In 2003, the Tanzanian government created guidelines for investigation and control of foodborne diseases and the regulatory institution is the Tanzania Food and Drugs Authority (TFDA) (Table 2) [325]. The main objective of TFDA is to regulate matters related to food quality and safety for consumers through the dissemination of the information related to causative agents, latency period [duration], principal symptoms, typical vectors, and prevention of poisoning as measures of public health protection [325]. Among several foodborne disease sources, MTs such as CTXs, TTXs, DA, and PSTs are described by TFDA. The creation of alert and monitoring programs is an effective way to prevent poisoning episodes caused by MT-contaminated seafood. 

### 3.4. Kenya

In order to reduce the cases of seafood poisoning caused by MTs, the Kenya Marine and Fisheries Research has carried out projects funded by governmental and non-governmental institutions for monitoring levels of HABs and their toxins (Table 2) in coastal waters and shellfish as well as the possible transfer in the trophic food web [326].Since October 2017, there is an ongoing project called: The occurrence and distribution of HABS in East and South Africa (BIOTOXINS Research Project] funded by National Commission for Science, Technology and Innovation (NACOSTI) at Mombasa Research Center [326]. This project will cover a period of 2 years, which is not enough for long-termmonitoring. In these coastal waters were reported to occur several species of diatoms: *Nitzschia* sp., *N. closterium, N. longisigma, N. sigma, Pseudo-nitzschia* sp. *Guinardia* sp., *G. striata, G.delicatula, Skeletonema* sp, *Leptocylindrus* sp., *Rhizosolenia* sp., *Cerataulina* sp., *Coscinodiscus* sp., *Thalassiosira* sp., *Corethron* sp., *C. criopilum, C. cenofemus* and *Chaetoceros* sp.; dinoflagellates: *Alexandrium* sp., *Dinophysis* sp., *D. caudata, Gambierdiscus* sp., *G. toxicus, Gonyaulax* sp., *Gymnodinium* sp., *Gyrodinium* sp., *Ostreopsis* sp., *Peridinium* sp., *Prorocentrum* sp., *Ceratium* sp., *C. fusus, C. furca, Noctiluca* sp., *N. scintillans*, *Protoperidinium* sp., *Scrippsiella* sp. and *S. trochoidea* [301,310]. Cyanobacteria were also reported: *Lyngbya* sp., *Oscillatoria* sp., *Fischerella epiphytica*, *Anabaena* sp., *Nodularia spumigena*, *Umezakia natans*, *Aphanizomenon flos-aquae, Microcystis aeruginosa and Trichodesmium* sp. [342]. 

### 3.5. Madagascar 

Madagascar is the country with more records of published data regarding MT occurrence (Figure 14) and consequently, many reported cases of seafood poisoning [36,47,49,343]. The seafood poisoning cases in Madagascar have been registered since 1930 mainly after the consumption of fish of the family *Sphyrnidae, Cacharinidae, Clupeidae* (herrings, sardines), and marine turtles species (*Eretmochelys imbricata* and *Chelonia mydas*) [36,47,49,343]. The main marine poisoning causative agents reported are CTXs, TTXs, and PlTXs [18,344] (Table 4). To reduce the number of seafood poisoning events, the MadagascarMinistry of Health has created a Seafood Poisoning National Control Program (Table 2) based on the setting of an epidemiological surveillance network, prevention of the communities through educational programs and the development of research on marine eco-environment [327].

### 3.6. Indian Ocean French Islands 

Mayotte, Europa, Banc du Geyser, Bassas da India, Glorioso, Juan de Nova, Reunion and Tromelin islands administratively make part in the French government but since they are in the Indian Ocean, were considered for the present study. In these islands, there are reports of the occurrence of HABs and cases of seafood poisoning linked to MTs. The reported HAB forming species include: dinoflagellates (*Prorocentrum lima, P. convacum, Ostreopsis ovata, Gambierdiscus toxicus, Alexandrium* spp.), cyanobacteria (*Hydrocoleum* sp., *Lyngbya majuscula, Phormidium* sp., *Leptolyngbya* sp. and *Oscillatoria* sp.) [70,300,317,319,345]. The recorded human intoxications were due to DSTs and TTXs [35,328] (Table 4). Centers of Disease for control and Preventing is the organization responsible for National Biomonitoring Program of toxins (PSTs) in these islands [35,328] (Table 2).

### 3.7. Mauritius 

In Mauritius there are registered cases of seafood poisoning caused mainly by CTXs [346] after the consumption of reeffish (*Lutjanus sebae*) [70,71,71] (Table 4). The Ministry of Ocean Economy, Marine Resources, Fisheries and Shipping of Mauritius is the institute responsible for themonitoring of HABs (Table 2) [347,348], developing several activities and reporting the principal vectors species involved in seafood poisoning, namely: fish (*Variola louti, Plectroponus maculatus, ceragidae, Vieille loutre, V. plate, V. cuisinier, Lutjanus gibbus, L. sebae, L. monostigmus, L. bohar, Anyperodon leucogramnicus, Harengula ovalis, Sphyraena barracuda, Synancela verrucose, Remora remora, Lactoria carnuta, Diodon hystrix*), turtles (*Eretmochelys imbricate*), crabs (*Carpillus maculatus*), sea-urchins (*Echinothrix* sp.) and bivalves (*Tridaena* sp.) [348].

HAB producers recorded in Mauritius include several dinoflagellates species (*Ostreopsis mascarenensis*, *Gambierdiscus toxicus* Adachi & Fukuyo, *Ostreopsis ovata* Fukuyo, *Ostreopsis siamensis*, *O. mascarenensis*, *Prorocentrum lima*, *P. concavum*, *P. hoffmanianum*, *Amphidinium* sp., *A. carterae*, *Coolia* sp., *Sinophysis* sp., *Gymnodinium* sp., *Gonyaulax* sp., and *Alexandrium* sp.), diatoms (*Pseudo-nitzschia* sp.) and cyanobacteria (*Phormidium* sp., *Oscillatoria* sp. and *Lyngbya* sp., *Phormidium* sp., *Oscillatoria* sp. and *Lyngbya* sp.) [308]. 

### 3.8. The Archipelago of Comoros

Published data of the archipelago of Comoros indicate the occurrence of *Gambierdiscus toxicus*, *G. yasumotoi*, *G. belizeanus*, *Prorocentrum arenarium*, *P. maculosum*, *P. belizeanum*, *P. lima*, *P. mexicanum*, *P. hoffmanianum*, *P. concavum*, *P. emarginatum*, *P. elegans*, *P*. sp., *Ostreopsis caribbeanus*, *O. mascarenensis*, *O. ovata* *O. heptagona*, *O. labens*, *O. siamensis*, *O. lenticularis*, *O. marinus*, *Cooliamonotis*, *C. tropicalis*, *Sinophysis microcephalus*, *S. canaliculate* and *Amphidiniopsis* sp. [10,300]. Suspected seafood poisoning episodes linked to MTs were registered in the archipelago of Comoros after the consumption of turtle Eretmochelys imbricate with symptomatology similar to CFP [26], suggesting the presence of CTXs (Table 4).

### 3.9. Somalia and Seychelles 

There are no published studies related to the occurrence of HABs and MTs in Somalia and Seychelles. While there are no published reports of HABs or MTs in Somalia and Seychelles waters, the proximity to other countries with such reports and currents in the area suggest that investigations are necessary to avoid potential seafood poisoning events [62].

### 3.10. Mediterranean and Red Sea (Djibouti, Eritrea, Sudan, Egypt)

Several research works related to MTs are carried out in the Red Sea but are very limited on the African coast. Saudi Arabia is the country with the most published studies related to the occurrence of HABs along the Red Sea [13,308,311,316,321,322,352,353]. The Dinoflagellates (*Alexandrium* sp., *Dinophysis* sp., *Prorocentrum* sp., *Pyrodinium* sp., *Gymnodinium* sp.), cyanobacteria (*Lyngbya* sp., *Oscillatoria* sp., *Trichodesmium* sp.) and diatoms (*Pseudonitzschia* spp.) are the most reported marine producer species [13,308,311,316,321,322,352,353]. The bacteria *Vibrio paraehemolyticus*, producer of TTX, was detected in shrimp (*Penaeus latisulcatus*) in the Suez Gulf [299]. MTs reported in the Red Sea, mainly the Egyptian coast, described in Table 3 and Table 4, include CTXs, TTXs, PSTs detected in puffer fish such as *Pleuranacanthus sceleratus* and *Lagocephalus sceleratus* [13,316,349,350,351,352,353]. Cases of seafood poisoning caused by CTXs and TTXs were reported, and according to the Poison Control Center, affiliated with Ain Shams University (Cairo, Egypt), CTXs are the third most responsible agents that induce food poisoning in Egypt [354]. Puffer fish poisoning has been recorded since ancient Egyptian times [42]. In Egypt, there is monitoring ofHABs in aquatic ecosystems since 1994 when Egypt became a member of the Convention on Biological Diversity although the Nature Conservation Sector, Egyptian Environment Affairs Agency and the Ministry of State for Environmental Affairs (Table 2) are focal points [330]. There are no reports of HABs and MT occurrence in coastal areas of Djibouti, Eritrea, and Sudan.

## 4. Final Considerations and Recomendations

African Indian Ocean and the Red Sea coasts have a subtropical and tropical climate, considered optimal for the development and transportation of several HAB-forming species, and consequently, the production of MTs. Paradoxically, studiesrelated to the occurrence and incidence of HABs and MTs are very limited, from South Africa to Egypt. From a few data available in this zone, most describe only the genus and not the full species, making it very difficult to evaluate the occurrence of the toxic species. The most reported HAB phytoplanktons in this region are cyanobacteria, followed by dinoflagellates, and diatoms as potential MT producers. Relative to MTs, the most reported and involved in seafood poisoning episodes include CTXs, PSTs, and TTXs. The scarcity of the data related to MTs suggests the need for further studies and the creation of specific monitoring programs of HABs, particularly for dinoflagellates and diatoms since these constitute the phytoplankton that produces more fatal MTs, though in recent years several genera of bacteria have been described as producers of a potent group of marine toxins, TTXs, which have already been detected on the African coasts of the Indian Ocean and Red Sea. The main MTs that must be monitored in shellfish are presented in Table 5. Analytical techniques such as LC-MS/MS are advised and recommended as determination and quantification methods due to their higher reproducibility, specificity, sensitivity and capacity to discriminate analogs of given toxins in the sample. The permitted limit of a toxin in shellfish can be adopted from other countries as an example to follow such as the EU region, USA, Japan, Australia, and New Zealand.

For the success of the MT monitoring programs, the integration and intercollaboration of environmental, public health and researches institutions and universities of the all African Countries of the Indian Ocean and the Red Sea is crucial.

## Figures and Tables

**Table 1:** Marine toxins and their symptoms, producers, permitted limit, detection methods, limit of detection/limit of quantification [LOD/LOQ] and toxicity equivalency factors [TEF] according to the European Food Safety Authority [EFSA].

Toxin (Syndrome)	Symptoms	Detection			Permitted Limit	Toxin (TEF)	Producer
		Methods	LOD, μgKg^−1^	LOQ, μgKg^−1^			
**OA and analogs (DSP)**	diarrhea, nausea, vomiting, abdominal pain and tumor formation in the digestive system [50]	BA [180,181]	160		0.16mg OA equivalents/Kg shellfish meat in EU region [182]	OA[**1.0**]	Dinoflagellates: *Prorocentrum* spp. [8], *Dinophysis* spp. [2,6,9,10,15,53,54] and *Phalacroma rotundatum* [55]
						DTX1[**1.0**]	
		EIA [183,184,185,186]	10–26	3–41			
						DTX2 [**0.6**]	
		LC-MS [183], -UVD [187]	15–30	1–50			
						DTX3 [**1.0; 1; 0.6**]	
**CTXs and analogs (CFP)**	vomiting, diarrhea, nausea, tingling, itching, hypotension, bradycardia. In extreme cases, death through respiratory failure in 30 min and 48 h after fish consumption [50]	BA [188,189]	0.16–0.560 P-CTX [190]		0.01 μg P-CTX-1 equivalents/kg of fish in USA [191]	P-CTX-1[**1.0**]	Dinoflagellates: *Gambierdiscus toxicus, Ostreopsis siamensis* and *Prorocentrum lima* [59]
		CTA [192,193,194]	~10^6^ - 0.039 C-CTX			P-CTX-2[**0.3**]	
						2,3-dihydroxy P-CTX-3C[**1.0**]	
		EIA [72,189,195,196,197,198,199]	-0.032 P-CTX				
		LC-MS/MS [67,70,71,74,200], -UVD [62,201,202]				C-CTX-1[**0.1**]	
**CIs**	non-specific symptoms such as gastric distress and tachycardia in humans [82]	BA	5.6–77 PnTXE		Not regulated	13-desmethyl SPX C[**1.0**]	Dinoflagellates: SPXs: *Alexandrium* spp. [1,76], GYMs: *Gymnodium* spp. [77], PnTXs: *Vulcanodinium rugosum* [78] and PtTXs: biotransformation from PnTXs via metabolic and hydrolytic transformation in shellfish [1,5,77,78,79]
		FPA [203]	80–85 13-SPXC				
		LC-MS/MS [79,204], - UVD [205]	0.8–20 13-SPXC/GYMA				
**PbTxs and analogs (NSP)**	nausea, vomiting, diarrhea, paresthesia, cramps, bronchoconstriction, paralysis, seizures in 30 min to 3 h [87]	BA [206]			800 μg BTX-2 equivalents/kg shellfish in USA [98], New Zealand, and Australia [99,100]	BTX-2, BTX-3, BTX2-B2 and S-deoxy-BTX-B2 [**same TEF**]	Dinoflagellate: *Karenia* spp. [4,16,87]
		CTA [192]	250 BTX-1				
		RB [108]	30BTX-3				
		EIA [207,208]	1 BTXs and	25 BTXs			
		LC – MS/MS [209]	0.2 – 2 BTXs				
**PTX and analogs**	No specific symptoms	MBA	-		160 µg OA equivalents./kg shellfish meat in EU region [210]	PTX [1,2,3,4,6 and 11][**1.0**]	Dinoflagellate: *Dinophysis acuta* [101]
		EIA [207]	-				
						PTX [7,8,9 and 2SA] and 7-*epi*PTX2 SA [<<**10**]	
		LC – MS/MS [211,212]	1				
**YTX and analogs**	No specific symptoms	BA			3.75 mg YTX equivalents/Kg shellfish meat in EU region [124]	YTX[**1.0**]	Dinoflagellate: *Protoceratium reticuatum* [4,109], *Lingulodinium polyedrum* [4] and *Gonyaulax polyhedral* [4]
		EIA [213]				1a-homoYTX[**1.0**]	
						45-hydroxyYTX[**1.0**]	
		LC-MS/MS [111]	0.017				
						45-hydroxy-1a-homoYTX[**0.5**]	
**AZA and analogs (AZP)**	nausea, vomiting, diarrhea and decreased reaction to stomach cramps, deep pain, dizziness, hallucinations, confusion, short-term memory loss, seizure [214]	BA [181]	0.05		0.16 mg AZA1equivalents/Kg shellfish in EU region [210]	AZA1[**1.0**]	Dinoflagellates: *Azadinium spinosum* [117] and *Protoperidinum crassipes* [118]
						AZA2[**1.8**]	
		LC-MS/MS					
						AZA3[**1.4**]	
						AZA4[**0.4**]	
						AZA5[**0.2**]	
**STX and analogs (PSP)**	Numbness in the face and neck; headache, dizziness, nausea, vomiting, diarrhea, muscular paralysis; pronounced respiratory difficulty; death through respiratory paralysis [215]	BA [216,217]			0.8 mg STX equivalent/Kg shellfish in EU region [210]	STX[**1.0**]	Dinoflagellates: *Alexandrium* spp. [2,3,7], *Gymnodinium catenatum* [3], *Pyrodinium bahamense* [3] and cyanobacteria *Trichodesmium erythraeum* [131]
						NSTX[**1.0**]	
		SBA [218]				GTX1[**1.0**]	
						GTX2[**0.4**]	
						GTX3[**0.6**]	
		CTA [192,219]				GTX4[**0.7**]	
						GTX5[**0.1**]	
		Antibodies Assay [220,221,222,223,224]				GTX[**0.1**]	
						C2[**0.1**]	
		Eletrophoresis [225]				C4[**0.1**]	
						de-STX[**1.0**]	
		LC-MS/MS [226,227,228,229]	23–42 STX			de-GTX3[**0.2**]	
						de-NSTX2[**0.2**]	
						de-GTX3[**0.4**]	
						11-hydroxy-STX[**0.3**]	
**DA and analogs (ASP)**	nausea, vomiting, diarrhea or abdominal cramps] within 24 h of consuming DA contaminated shellfish and/or neurological symptoms or signs [confusion, loss of memory or other serious signs such as seizure or coma] occurring within 48 h	BA [230]	40		20 mg DA equivalents/Kg shellfish in EU region [210]		Diatoms: *Pseudo-nitzschia* spp. [126] and red algae: *Chondria armata* [127].
		(a) ASP- EIA [184,231]	0.003	0.01			
		SPR [232]	20				
		RB [233,234,235]	20				
		Capillary electrophoresis [236,237,238]	0.15 -1				
		LC -MS/MS [211,239,240], UVD [241,242]	0.015				
		TLC [243]	10				
**TTX and analogs**	Vomiting, strong headache, muscle weakness, respiratory failure, hypotension and even death in hours [244]	BA [144,245,246,247]	1.1 [247]		2 mg TTX equivalents/Kg shellfish in Japan [248]	S/R 11-norTTX-[6]-ol[0.19/0.17]	Bacteria: *Serratia marcescens, Vibrio* spp. [83], *V. Aeromonas* sp. [138], *Microbacterium, arabinogalactanolyticum* [139], *Pseudomonas* sp. [140], *Shewanella putrefaciens* [141], *Alteromonas* sp. [142], *Pseudoalteromonas* sp. [143], and *Nocardiopsis dassonvillei* [144]
		RB [249]	2–4.10^−3^TTX				
						4-*epi*TTX[**0.16**]	
		EIA [245,246,247,250,251,252,253,254,255,256]	0.002/mL [255], 0. 0001/mL [253]				
		TLC [139,257]	2 [257]			4,9-anhydroTTX[**0.02**]	
		GC-MS [28,258,259]	500	1000 [258]			
						5,6,11-deoxyTTX[**0.01**]	
		LC-MS/MS [260,261,262,263,264] – FLD [265]	0. 00009?-24.5 [260,261,262,263,264]	40 [265] – 100 [265]			
**PlTX**	Vasoconstriction, hemorrhage, myalgia, ataxia, muscle weakness, ventricular fibrillation, ischemia and death [266,267] and rhabdomyolysis [268]	BA			Not regulated toxin but proposed value is 0.25mg PlTX equivalent/Kg shellfish in EU region [269]	PlTX[**1.0**]	Zoanthids: *Palythoa* spp. anddinoflagellates: *Ostreopsis ovata*. [153,154,155] and possibly cyanobacteria: *Trichodesmium* sp. [156]
		Hemolysis assay [270]	1.6				
		CTA [107]	50				
						streocin-D[**0.4–1.0**]	
		EIA [254]	1/mL				
		LC-MS/MS [204,271]–FLD and–UVD [272]	2,5.10^−5^–0, 50.10^−5^				
**MC**	liver hemorrhage within a few hours of an acute dose and death [273]	LC-MS [167,274,275,276] and EIA [277]			Tolerable daily intake: 0.04 μg/kg of MC body weight/day [278]		Cyanobacteriaof genus: *Pseudoanabaena, Phormidium, Spirilia* [164], *Leptolyngbya, Oscillatoria, Geitlerinema* [165], *Trichodesmium* [166] and *Synechococcus* [167]
**ANTX and HANTX**	Hypersalivation, diarrhea, shaking and nasal mucus discharge [279], respiratory arrest and death [280]	RB and GC/MS [281,282]					Cyanobacteria: *Hydrocoleum lyngbyaceum* [177]
**AT and DAT**	Contact dermal: dermatitis initiating with erythema and burning sensations, appearing a few hours after exposure, gave way to blister formation and deep desquamation, lasting up to several days [283,284] and consumption of contaminated seafood; burningsensation in the mouth and throat, vomiting and diarrhea [285]	LC-MS/MS [286]					Algae *Gracilaria coronopifolia* [172] and cyanobacteria *Lyngbya majuscula* [171]
**LA, LB, and LC**							Cyanobacteria *Lyngbya majuscule* [174]
**ATX and analogs**	No specific symptoms	LC [287]					Cyanobacteria: *Lyngbya majuscula* [179]
**JCD and analogs**	No specific symptoms	LC, TLC and [288]					Cyanobacteria: *Lyngbya majuscula* [176]
**KTX and analogs**	No specific symptoms	LC [173]					Cyanobacteria: *Lyngbya majuscula* [173]
**CYN and analogs**	Gastroenteritis [289]	LC-MS/MS [290],–PDAD [291]	1 [292]–200 [293]				Cyanobacteria: *Cylindrospermopsis raciborskii* [175]
		EIA [294]					

**Toxins**: DA—domoic acid, DTX, CTX -ciuatoxin, AZA—azaspiracid, CI—cyclic imines, PTX—pectenotoxin, YTX—yessotoxin, STX—saxitoxin, OA—okadaic acid, BTX—revetoxin, PlTX—palytoxin, TTX -tetrodotoxin, MC—microcystin, ANTX—anatoxin, HANTX—homoanatoxin, LA, LB and LC—lyngbyatoxins A, B and C respectively. ATX—antillatoxin, KTX—kalkitoxin, CYN—cylindrospermopsins AT—aplysiatoxin, DAT—debromoaplysiatoxin, JCD—jamaicamides, **Syndrome**: PSP—Paralyc Poisoning, DSP—Diarrheic Shellfish Poisoning, ASP—Amnesic Shellfish Poisoning, AZP—Azaspiracid Shellfish Poisoning, CFP—CiguateraShellfish Poisoning, NSP—Neurologic Shellfish Poisoning, **Detection methods**: CTA—Cytotoxicity assay, EIA—Enzyme-ImmunoAssay, SPR—Surface Plasmon Resonance, RB—Receptor-based, GC—Gas Chromatography, BA—Bioassay; UVD—Ultra Violet Detection; LC—Liquid Chromatography and MS—Mass Spectroscopy, FPA—Fluorescence Polarization Assay, TLC—Thin Layer Chromatography, SBA—Saxitoxin Binding Assay, PDAD—photo diode array detection.

**Table 2:** MT monitoring scenario of the African countries of the Indian Ocean and the Red Sea.

Country	Monitored MT	Permitted Limit, mgKg^−1^ Shellfish	Detection	Laboratories for Toxin Analysis	Reference
South Africa	PST	0.8 STX		Research centers and Universities	[324]
	OA, DTX1-2, PTX1-2	0.16 mg OA	LC-MS/MS		
	YTX, 45 OH YTX, homo YTX, and 45 OH homo YTX	8 mg YTX	LC-MS/MS		
	AST	20 mg DA			
	AZA1-3	0.16 mg OA	LC-MS/MS		
Mozambique	N.D.	N.D.	N.D.	N.D.	N.D.
Tanzania	CTX, TTX, AST	N.D.	Symptomology and vectors	N.D.	[325]
Kenya	MT producers [HAB]	N.D.	N.D.	Mombasa Research Center	[326]
Madagascar	N.D.	N.D.	Educational programmes	Researches centers and Universities	[327]
French Islands	N.D.	N.D.	N.D.	Researches centers	[35,328]
Mauritius	N.D.	N.D.	N.D.		[324]
Comoros	N.D.	N.D.	N.D.	N.D.	
Somalia and Seychelles	N.D.	N.D.	N.D.	N.D.	
Eygpt	N.D.	N.D.	N.D.	Poison Control Center, Ain Shams University	[329,330]
Djibouti	N.D.	N.D.	N.D.	N.D.	
Eritrea	N.D.	N.D.	N.D.	N.D.	
Sudan	N.D.	N.D.	N.D.	N.D.	

N.D.—No Data.

**Table 3:** Geographic occurrence MT per country, MT producer, and MT vector along African countries of the Indian ocean and red sea coasts. TX - toxin.

Toxin	Date	Location	Toxin Producer	Determination Method	Toxin Vector	TX Concentration, (mg TX Equivalents per Kg Shellfish Meat)	Cell/Extract Toxicity	Reference
PSTs	1999	South Africa	*Alexandrium catenella*	AOAC mouse bioassay	*Haliotis midae*	0. 01609 STX		[22]
	1998–2002	South Africa: Yzerfontein,	*Alexandrium catenella*	HPLC-FLD	-	-	4.8 pg STX eq cell^−1^	[334]
			*Alexandrium tamiyavanichi*				0.14 pg STX eq cell^−1^	
	2003–2004	South Africa: Cape Town	*Alexandrium minutum*	LC-FD and HILIC-MS/MS	-	-	0.65 pg GTX cell^−1^	[309]
	2012–2014	Central Red Sea	*Pyrodinium bahamense, Ceratium* sp., *Alexandrium* sp. and *Protoperidinium* spp.	ELISA	-	-	>> 0.4 ng mL^1^	[349]
DSTs	2000	Europa Island Mozambic channel, France]	*Prorocentrum arenarium*	FR3T3 fibroblast	*-*	-	IC_50_ = 0,1 µg OA ml^−1^ and 50 µg extract ml^−1^	[11]
				PPIA				
				HPLC-FD				
				HPLC-MS			22 ng OA/mg of extract	
	2001	Lagoons of La Reunion Mayotte and Mauritius Islands	*Prorocentrum* *lima*	PPIA	-	-	IC_50_ 1.3–25 mg/mL onon fibroblast;6261.3 ± 156.5 − 128.3±17.2 ng eq OA/mg crudeextract	[328]
	2002–2018	South Africa:Abalgold	-	-	*Haliotis asinina*	-	-	[324]
	2008	South Africa: Saldanha Bay and Lambert’s Bay	*Dinophysis acuminata*	LC-MS/MS	*Crassostrea gigas*	0.267 OA		
					*Choromytilus meridionalis*	0.012 OA		
CTXs	2001	Mauritius: Nazareth, Saya de Malha and Soudan	-	HPLC-MS/RLB, Mongoose feeding test, and MBA	*Lutjanus sebae* and *Lutjanus* *Lab*	Qualitative analysis	-	[71]
	2002	North of the Republic of Mauritius, Banks fishery	-	HPLC-MS/RLB	*Lutjanus sebae*		-	[70]
	2012–2013	Central Red Sea	*Gambierdiscus belizeanus* and *Ostreopsis* spp.	Mouse neuroblastoma cell-based assay	-	-	6,50–1,14.10 ^−5^ pg P-CTX^−1^ eq. cell^−1^	[350]
	2013	Madagascar: district of Fenoarivo Atsinanana	*Gambierdiscus* spp.	CBA	*Carcharhinus leucas*	0.083 P-CTX-1	-	[20]
				MBA		0. 09272 P-CTX-1		
				LC-ESI-HRMS		0. 01628 P-CTX-1		
				MBA		752 MU/g		
PlTXs	1994	Madagascar: Antalaha District	*Ostreopsis siamensis*	MBA	*Herklotsichthys quadrimaculatus*	0. 00045 PTXs/fish [head and esophagus]		[18]
				Hemolysis assays		0. 00002 PTXs/fish [head and esophagus]		
				Cytotoxicity tests		0. 00000005/fish [head and esophagus]		
				MS				
	1996	Mauritius: Rodrigues Island	*Ostreopsis mascarenensis*	HPLC-diode array detector, Nanoelectrospray ionization quadrupole time-of-flight and HPLC-ESI-MS/MS analysis	-	-		[14,160]
				Hemolysis assays			8.00 ± 0.01 ng PTX mL^−1^	
				Cytotoxicity Assay			IC50 = 10 μM against human H460 lung cancer cells	
	2008	South Africa: Saldanha Bay and Lambert’s Bay	*Dinophysis acuminata*	LC-MS/MS	*Crassostrea gigas*	0.267 OA		
					*Choromytilus meridionalis*	0.012 OA		
DA cultures	2012	South Africa: Algoa Bay	*Pseudo-nitzschia multiseries*	ELISA	-	-	0.076 pg DA cell^−1^–0.098 pg DA cell^–1^	[12]
				LC/MS–MS			0.086 pg DA cell^–1^–0.086 pg DA cell^–1^	
TTXs	1990–1991	Egypt: Suez City, in the northwestern part of the Red Sea		TLC, electrophoresis, UV, GC–MS	*Pleuranacanthus* *sceleratus*			[316]
						752 MU/g		
				MBA				
	1998	Madagascar: Nosy Be Island -	-	MBA		16 MU/g		[41]
	2002–2003	Egypt: Gulf of Suez		MBA	*Lagocephalus sceleratus*	3950 MU/g		[351]
	2013	Reunion Island		MBA and LC-MS/MS	*Lagocephalus sceleratus*	17 TTX	-	[35]

**Table 4:** Seafood poisoning episodes caused by MTs, observed effects/Symptoms, fish or shellfish consumed and victim number affected along African countries of the Indian Ocean and Red sea coasts. TX - Toxin

Local	Date	Seafood	Observed Effects/Symptoms	TX	Detection Method	TX Concentration, (mg TX Equivalents/Kg Shellfish Meat)	Victim Number	Reference
Comoros islands: Ndrondroni	24 December 2012	*Eretmochelys imbricata* (turtle)	Itching, Asthenia, Vomiting, Abdominal pain, Rash Myalgia Shortness of breath, Nausea Itching of the mouth/throat, Fever, Diarrhea Vertigo, Paresthesia, Dysphagia Mouth burn Sore throat, Erectile dysfunction	-	-	-	49 suspected cases and 8 probable cases, age range [0–40 years], 1 death	[26]
North-eastern coast of Madagascar	December 1994	Turtle	Nausea, vomiting, dysphagia, acute stomatitis	-	-	-	60 persons with poisoning attack rate were 48% with a lethality of 7.7%	[47],
Madagascar: district of Fenoarivo Atsinanana	November 2013	Carcharhinus leucas (shark)	Paresthesia of the extremities, dysesthesia, and reversing sensitivity of hot and cold accompanied by a headache, dizziness, and arthralgia between 2 and 12h after ingestion	CTXs	MBA	0.083 P-CTX-1	124 people, 9% deaths	[20]
					CBA	0. 09272 P-CTX-1		
Madagascar: Antalaha District	January 1994	*Herklotsichthys quadrimaculatus* (Fish)	Malaise, uncontrollable vomiting, diarrhea, tinglings of extremities, delirium and death	PlTXs	MBA	0. 00045 PTXs/fish [head and esophagus]	Death of one adult	[18]
					Hemolysis assays	0. 00002 PTXs/fish (head and esophagus)		
					Cytotoxicity tests	0. 00000005/fish (head and esophagus)		
					Mass spectroscopy	-		
Madagascar: Nosy Be Island	July 1998	-	-	TTXs	MBA	16 MU/g (no data to covert to mg/Kg)	4 people, one death	[41]
Madagascar: Manakara district	November 1993	*Carcharhinus amboinensis* [shark]	Deep coma and death, body rigidity due to loss of cerebral function, myosis, mydriasis, convulsions, Respiratory distress due to acute pulmonary edema, cardiovascular collapse, bradycardia, gengivorrhagia Dehydration, paresthesia on fingertips and toes, dizziness, pruritus, narcosis, faintness, hyperthermia, ataxia asthenia, dehydration, cephalalgia, diarrhea, epigastralgia, laryngeal distress	CTXs	Ciguatera poisoning Symptomology	-	500 people, 20% deaths	[21]
South Africa: Cape Town	May 1978	*Choromytilus meridionlis* [Mussel]	Paraesthesia of en fingers/hands, Circumoral paresthesia, paranesthesia of toes/feet, Vertigo, Floating sensation, Ataxia, Weakness of upper, Weakness of lower limbs and Dysarthria A headache	PSTs	MBA	72.83 STX	17 people, no deaths	[39]
South Africa: Natal coast	December 1957	*Mytilus* *meridionalis* [Mussel]	peculiar lightness of the body, with a tingling around mouth, finger, and toes; no moving; feeble inarticulate noise;	PSTs	MBA	0.04 STX	5 people and one cat	[40]
South Africa: Table and False Bays	1888	Donax serra [Mussel]	-	-	-	-	-	[37]
South Africa: Cape Town	April 1948	*Donax serra* [Mussel] and *Chloromytilus* *meridionalis* [Mussel]	-	-	-	-	One death	
South Africa: Natal coast	December 1957	*Perna perna* [Mussel]	-	-	-	-	5 people, one death	
South Africa: Cape Town a	May 1958	*Chloromytilus meridionalis* [Mussel]	-	-		-	One death	
Reunion Island	September 10th, 2013	*Lagocephalus sceleratus* [fish]	peri-oral paresthesia, weakness of both lower limbs, paresthesia all over the body, headache, dyspnea, nausea and vomiting, blurring of vision, and vertigo	TTX	MBA	Liver: 17 TTX Flesh: 5 TTX	10 people	[35]

**Table 5:** Recommended marine toxins to be monitored and suggestion of permitted limit to be used.

Toxin	Syndrome	Permitted Limit, mgKg^−1^	To be adopted from
STX	PSP	0.8 STXeq	EU region
CTX	CFP	0.00001 P-CTX-1eq	USA
YTX	-	3.75 YTXeq	EU region
PTX	-	0.16 OAeq	EU region
TTX	-	2 TTeq	Japan
DA	ASP	20 DAeq	EU region
OA	DSP	0.16 OAeq	EU region
AZA	AZP	0.16 AZAeq	EU region
PlTX	-	0.25 PlTXeq *	EU region
PbTx	NSP	0.8 TX-2 eq	USA, New Zealand, and Australia

* This toxin is not monitored and 0.25 PlTXeq was proposed in the first meeting (Cesenatico, Italy, 24–25 October 2005) of the working group on Toxicology of the national reference laboratories [NRLs] for Marine Biotoxins.
